# The Effect of the Husbandry System and Cortisol Status on the Response of Water Buffalo Calves to Vaccination with the *Brucella abortus* Vaccine RB51

**DOI:** 10.3390/vetsci13070612

**Published:** 2026-06-25

**Authors:** Nadia Piscopo, Esterina De Carlo, Anna Cerrone, Domenico Vecchio, Michele Napoletano, Agata Campione, Chiara Denise Ambra, Michael J. D’Occhio, Marco Esposito, Alessio Cotticelli, Tanja Peric, Giuseppe Campanile

**Affiliations:** 1 Department of Veterinary Medicine and Animal Productions, University of Naples Federico II, 80137 Naples, Italy; nadia.piscopo@unina.it (N.P.); giuseppe.campanile@unina.it (G.C.); 2 Experimental Zooprophylactic Institute of the South, National Reference Centre on Water Buffalo Farming and Productions Hygiene and Technologies, 80100 Naples, Italy; esterina.decarlo@izsmportici.it (E.D.C.); anna.cerrone@izsmportici.it (A.C.); domenico.vecchio@izsmportici.it (D.V.); michele.napoletano@izsmportici.it (M.N.); agata.campione@izsmportici.it (A.C.); chiaradenise.ambra@izsmportici.it (C.D.A.); 3 School of Life and Environmental Sciences, The University of Sydney, Sydney, NSW 2006, Australia; michael.docchio@sydney.edu.au; 4 Campania Region-Prevention and Veterinary Public Health Unit, 80100 Naples, Italy; marco.esposito@regione.campania.it; 5 Human Sciences Department, Link Campus University, 00165 Rome, Italy; 6 Department of Agricultural, Environmental and Animal Science, University of Udine, 33100 Udine, Italy; tanja.peric@uniud.it

**Keywords:** vaccine RB51, buffalo farms, management, cortisol, antibody response

## Abstract

Brucellosis negatively impacts animal and human health and causes major economic losses. The present study examined the relationship of husbandry system (insufficient, acceptable, optimal) and cortisol status on the response of buffalo calves to vaccination with the brucellosis vaccine RB51. Primary vaccination (1st) was at 6 months and secondary vaccination (2nd) at 12 months. Cortisol was ascertained in samples of hair and the antibody response to vaccination was measured in blood. Excretion of the bacterium *Brucella abortus* was determined in urine and feces. Buffalo calves in the insufficient system had greater (*p* < 0.05) cortisol (4.7 ± 0.5 pg/mg) at 1st vaccination than calves in the optimal system (2.8 ± 0.3 pg/mg). Cortisol was lower at the 2nd vaccination for all three systems which did not differ. Anti-RB51 antibodies were present in 28/30 buffalo after 2nd vaccination. The conditional *R*^2^ of 0.689 indicated that both husbandry system and cortisol status strongly influenced the response to vaccination with RB51. No shedding of *Brucella* spp. was observed. The study has shown that husbandry system and cortisol status influence the response of buffalo calves to vaccination with RB51.

## Simple Summary

*Brucella abortus* causes brucellosis, which negatively impacts human and animal health and inflicts significant economic losses to livestock farms. This study examined the effect of husbandry system and level of stress measured by cortisol status on the response in buffalo calves to vaccination with the RB51 vaccine. After secondary vaccination, 28/30 buffalo showed an immune response to vaccination which was influenced both by husbandry system and cortisol status. The study has shown that husbandry and level of stress need to be carefully managed to optimize the response in buffalo calves to vaccination with RB51.

## 1. Introduction

The bacterium *Brucella abortus* is the causative agent for brucellosis, a disease of global significance given its presence in 160–170 countries [1,2]. Brucellosis is a highly contagious zoonotic disease [1] that infects many species including production, domestic, recreational and wild animals [3]. It is primarily a reproductive disease in females and causes abortion, still birth and retained placenta [4]. Hence, it has major animal welfare and economic consequences [5]. Water buffalo (*Bubalus bubalis*) are particularly susceptible to brucellosis which represents a major threat to the global population of around 150 million water buffalo. Buffalo are an essential source of affordable milk and meat in many countries and provide high-value animal-derived products in other countries [6]. The Italian water buffalo population exceeds 400,000 animals [7] with about 70% located in Southern Italy. Before 2010, the seroprevalence of buffalo brucellosis was much higher than during 2010–2020 (20.8% vs 9.7%) [2]. Despite efforts to control brucellosis in Italy it continues to impact both animals and humans.

Strategies to manage brucellosis include prevention, control, and eradication [8]. These approaches are based on evaluating hygiene and sanitation (biosecurity), the “test-and-slaughter” approach, and vaccination [8]. Brucellosis vaccination with the *Brucella abortus* strain 19 has been the reference vaccine in buffalo worldwide [9]. More recently, an alternative vaccine containing the *Brucella abortus* strain RB51 has been used successfully to control brucellosis outbreaks in several countries [4,9]. In cattle, RB51 vaccination prevented *Brucella abortus* infection and abortion in controlled experimental trials and under field conditions [10,11,12,13]. The RB51 vaccine has been used in the Campania Region of Southern Italy since 2007 and is now the preferred method for brucellosis control. It has become clear that the use of RB51 vaccination in water buffalo requires strict adherence to vaccination protocols [14]. The response to RB51 vaccination is influenced by animal management and husbandry, nutrition, and overall compliance with animal welfare standards [15].

The welfare of animals can be assessed using animal-based measures and measuring endocrine status [16]. The hypothalamic–pituitary–adrenal gland (HPA) axis is a key pathway that links stress and immunity, and chronic stress can dysregulate immune function [17,18]. Chronic stress is associated with elevated cortisol which suppresses the immune system [19]. In a recent review it was concluded that the environment experienced by production animals at a young age can have a major influence on the development of a robust immune system [20]. Based on this information, it was considered important to examine the effect of husbandry system and cortisol status on the response to RB51 vaccination in water buffalo calves. The findings would help to inform the husbandry system that is associated with an optimal immune response to vaccination with RB51 in young water buffalo. This would represent a further advance in the control and eradication of brucellosis in buffalo.

## 2. Materials and Methods

All experimental procedures were performed according to the European Directive 2010/63/EU and the Italian Legislative Decree No. 26 dated 4 March 2014 and received institutional approval from the Ethical Animal Care and Use Committee of the University of Naples “Federico II” (Protocol No. 0127143-2024).

### 2.1. Animals and Sampling

The study was carried out at three commercial buffalo dairy farms located in the province of Caserta (Southern Italy) according to the ‘Plan for the eradication of brucellosis and tuberculosis’ of the Campania Region (D.G.R.C. n. 104/2022). The Campania Region is a cluster area for brucellosis. At each farm, 10 female Italian Mediterranean buffalo calves received a primary (1st) vaccination against *Brucella abortus* RB51 at 198 ± 4 days and secondary (2nd) vaccination at 363 ± 2 days. The vaccine was produced by CZ Vaccines (batch no. 2405307, CZ VACCINES, S.A.U. A Relva, Torneiros s/n, 36410 O Porriño, Pontevedra, Spain). Each dose contained 10–34 × 10^9^ CFU of *Brucella abortus* strain RB51. The vaccine was stored at 2–4 °C and transported to study sites in refrigerated containers until the time of use. The vaccine (6 mL) was administered s.c. in the prescapular region by official veterinarians. No adverse events were observed in any of the animals enrolled throughout the study. Study sites were enrolled by evaluating the standard of husbandry using the ClassyFarm system for free-stall dairy buffalo [21]; as the study was designed as an observational field investigation reflecting the real management conditions of commercial farms, no control group was included. ClassyFarm is the Ministry of Health’s surveillance system for monitoring livestock farms and characterizing them according to risk.

The ClassyFarm checklist consists of a survey questionnaire covering 103 hazards (items) with multiple-choice answers, of which 15 relate to biosecurity and 88 to animal welfare. These are in turn divided into Area A (Farm Management and Personnel, 32 items), Area B (Facilities and Equipment, 30 items), and Area C (Animal-Based Measures (ABMs), 17 items), and Area D (Major Risks and Alarm Systems, 9 items). Each item is structured to be assessed using a grid with one or two risk thresholds and corresponding two or three response levels: Insufficient; Acceptable; Optimal. Each item has a different weight [22] and the overall animal welfare score of the farm is calculated using a 50% contribution by farm management, staff training, and housing systems, with the remaining 50% by animal-based measures [23]. The scoring system is based on a risk analysis mediated by a predefined algorithm, the weights of which were determined through an expert opinion elicitation carried out using the Delphi method [22]. This method correlates the results derived from the environmental inputs recorded with the indicators of animal adaptation to the farming environment, as determined by the ABMs, and thus establishes a score expressed as a percentage for each individual area and for the entire checklist. The overall score is expressed on a scale from 0% (lowest animal welfare) to 100% (highest animal welfare).

Based on the ClassyFarm check-list results [24], the three farms were classified into three animal welfare risk categories:

- Insufficient: poor condition of animals with negative implications for health and stress status (score < 70%).
- Adequate: fair conditions under which there is less likelihood that animals will experience negative welfare consequences (score 70–90%).
- Optimal: good conditions with the possibility for animals to live positively (score > 90%).

To obtain an endocrine index of welfare status, cortisol was measured in hair samples taken from each animal by the shave/re-shave technique [25]: at the time of 1st vaccination (T1) and 21 days later (T2) and at the time of 2nd vaccination (T3) and 21 days later (T4). Urine and fecal samples were collected from each animal on the day of 1st and 2nd vaccination and every 3 days for 15 days after each vaccination. Blood samples were obtained from the jugular vein on Days 0, 7, 14 and 21 at each vaccination.

### 2.2. Laboratory Analyses

Concentrations of cortisol (pg/mg) in hair samples were measured using a validated radioimmunoassay [26].

Urine samples were utilized for bacteriological analysis and molecular investigations according to established Standard Operating Procedures of the European Union Reference Laboratory (EURL) for Brucellosis, *Brucella* culture, and genus identification [27]. Fecal samples were processed according to an established procedure [28]. Presumptive colonies of *Brucella* spp. were evaluated using the oxidase (ThermoFisher Scientific™, Oxoid, UK), catalase (BioMerieux^®^, Campus De L’Etoile, 100 Allée Louis Pasteur, 69280 Marcy L’Etoile, France), and urease tests (BioMerieux^®^, Campus De L’Etoile, 100 Allée Louis Pasteur, 69280 Marcy L’Etoile, France) and further identified using the Vitek 2 System (VITEK^®^ 2 Compact, BioMerieux^®^, Campus De L’Etoile, 100 Allée Louis Pasteur, 69280 Marcy L’Etoile, France). Molecular investigations on urine and feces were carried out using real-time PCR and followed the Standard Operating Procedure ‘*Brucella* real time PCR’ of the EURL for Brucellosis and the OIE Manual of Diagnostic Tests and Vaccines for Terrestrial Animals [29]. Blood anti-RB51 antibody titres were measured using a complement fixation test that followed the recommendations of the National Reference Centre for Brucellosis (CNRB) at the Istituto Zooprofilattico Sperimentale dell’Abruzzo e del Molise (IZSAM). *Brucella* spp. antigen assessment was carried out in accordance with the recommendations of the *World Organisation for Animal Health Terrestrial Manual*, Chapter 3.1.4, Paragraph B2.3.1 for the buffered Brucella antigen tests and paragraph B2.4.3 for the complement fixation test [30]. The immunological endpoints were day 21 after both 1st and 2nd vaccination. Laboratory analyses for cortisol and antibody titre measurements were blinded to the husbandry system.

### 2.3. Statistical Analyses

Statistical analyses were performed in R version 4.4.3 for Windows 11. The normality of the dependent variables was tested using the Shapiro–Wilk test, and homogeneity of variance was assessed with Levene’s test via the car package. Log transformation was applied to meet the normality assumption. Antibody titres were expressed both as log-transformed continuous variables, as reciprocal of serum dilutions, and as binary values. To assess the combined effects of sampling times and husbandry systems on cortisol concentrations, a linear mixed-effects model was fitted using the lmer() function from the lme4 package. The model included the fixed effects of husbandry system, sampling time, and their interaction, with a random intercept for each animal to account for intra-individual variability. To explore pairwise differences in cortisol concentrations between husbandry systems and sampling times, estimated marginal means were calculated using the emmeans package. Tukey-adjusted comparisons were performed to control for multiple testing. To evaluate the relationship with antibody titres (as continuous and binary dependent variables) linear and logistic regressions were respectively fitted as follows:

- Linear mixed effect model: cortisol concentrations, sampling time and husbandry system (and their interaction) were the fixed effects and a random intercept for each animal was also included to account for intra-individual variability;
- Logistic mixed effect structure encompassing only cortisol concentrations, sampling time and husbandry system as fixed effects.

For mixed models, marginal and conditional *R*^2^ values were computed using the r.squaredGLMM() function from the MuMIn package. Model performance was assessed using McFadden’s pseudo-*R*^2^, the area under the receiver operating characteristic curve (AUC; computed with the pROC package), and classification accuracy at the default probability threshold of 0.5. Unless otherwise stated data are mean ± standard error (SE).

## 3. Results

Using the ClassyFarm system, the three farms had the following animal welfare scores: insufficient, 64%; adequate, 78%; optimal, 95%. Concentrations of cortisol in hair were log-transformed for parametric modelling. The fixed effects of the husbandry system (*p* < 0.05), sampling time (*p* < 0.01) and their interaction (*p* < 0.10) influenced hair cortisol concentrations. Animals raised in the optimal system tended (*p* = 0.09) to have lower average cortisol concentrations (1.8 ± 0.1 pg/mg) than animals raise in the insufficient (2.7 ± 0.3 pg/mg) and adequate (2.4 ± 0.2 pg/mg) systems, which did not differ. Cortisol was different among the husbandry systems only at T1 and T2 (Table 1, Figure S1).

A descriptive relationship between cortisol concentration (pg/mg, *n* = 30 animals per quartile) across each sampling time and husbandry system, and the anti-RB51 antibody titre (as reciprocal of serum dilutions), is shown in Table 2. Across cortisol quartiles, an inverse proportionality emerged, with higher cortisol concentrations consistently associated with lower geometric mean antibody titres.

The relationship between hair cortisol and antibody titre was further investigated by fitting a linear regression model with cortisol concentration (log-transformed), sampling time, and husbandry system (and their interaction) as fixed effects, and antibody titre (log-transformed) as the dependent variable, the animal was the random intercept (Table 3). The mixed-effects structure highlighted that immune function was strongly influenced by both stress (based on cortisol), husbandry system and sampling time, as shown by the conditional *R*^2^ (0.689).

The same approach was adopted, including the antibody titre as a binary dependent variable in logistic regression models. Husbandry system, sampling time, and cortisol, explained a substantial proportion of the variation in immune responsiveness (*R*^2^ = 0.389, Table S1).

The antibody titre across the three husbandry systems was different 21 days after 1st vaccination. In the adequate system, 10% of calves showed an antibody response with a titer of 1:8, while in the insufficient and optimal systems the response was 50% and 40%, respectively (*p* < 0.01). Seroconversion started from titres of 1:8 in the latter two systems and reached titres of 1:16 in the optimal system and 1:32 in the insufficient system.

After 2nd vaccination, an antibody response occurred in all animals in the insufficient (titres 1:32 to 1:128) and optimal (titres 1:32 to 1:256) husbandry system and in 80% of animals in the adequate husbandry system (titres 1:32 to 1:256).

Bacteriological analysis showed no shedding of *Brucella* spp. or the RB51 vaccine strain in urine and feces.

## 4. Discussion

In the present study, buffalo calves in the optimal husbandry system tended to have lower and more narrowly distributed hair concentrations of cortisol compared with calves in the insufficient and adequate systems. The high marginal *R*^2^ with linear and logistic modelling indicated that half of the variance in antibody titres was explained by cortisol status, sampling time and husbandry system, indicating a strong association with the humoral antibody response. Including animal as a random effect improved the model fit, with the conditional *R*^2^ (0.689) showing that between-animal variability was also a contributing factor to the humoral response. Overall, the findings support a strong association between cortisol status (chronic stress) and antibody titre, that is influenced by husbandry system, sampling time and animal. The immunosuppressive effect of chronic stress is well documented in humans [19] but is under-investigated in buffalo [31]. To our knowledge the present study is the first to report an association between hair cortisol and the immune response to vaccination against RB51 in buffalo.

Under controlled experimental conditions, all buffalo calves showed a serological response to 1st and 2nd vaccination with RB51 [14]. In the present study, 33% of buffalo had detectable anti-RB51 antibody titres after 1st vaccination and 93% had titres after 2nd vaccination. The present findings reflect the variability that can occur in the immune response under production conditions. This highlights why there remains a need to further refine vaccination protocols against brucellosis. Notwithstanding the apparent variability in the immune response among buffalo, the lack of the RB51 vaccine strain in fecal and urine samples was a promising finding.

Hair cortisol can be used as an indicator of the allostatic status of animals which results from cumulative stressors. At 1st vaccination, calves in the insufficient husbandry system had the highest concentration of cortisol. This was presumably due to the stress of weaning combined with inadequate husbandry. Weaning was associated with elevated cortisol in dairy calves [32].

At 2nd vaccination, hair cortisol was lower compared with 1st vaccination for all three husbandry systems. There were no differences between systems at 2nd vaccination. The decline in cortisol from 6 to 12 months was likely due to a combination of recovery from the stress of weaning, adaptation to the respective husbandry systems, and age [33,34,35]. The literature would suggest that age may have been the overriding factor in the decline in cortisol from 6 to 12 months [33,34,35]. The inclusion of animals as a random effect in the statistical analyses showed that between-animal variability was also a factor in the antibody response to RB51 vaccination.

The present study had limitations in methodology. The three husbandry systems were not replicated and there may have been farm-specific features that were not recorded. Notwithstanding, the ClassyFarm checklist has a broad set of indicators, and we are confident that the classification of the three husbandry systems was sound. The study was intentionally carried out at commercial farms to reflect real field conditions. This limited the number of animals that could be included and may restrict the extent to which the findings can be generalized.

## 5. Conclusions

The present study is the first to show an association between farm-level husbandry system, hair cortisol concentration used as an index of allostatic status, and the humoral response to RB51 vaccination in buffalo calves. The findings should be interpreted as hypothesis-generating given that each husbandry category corresponded to a single farm and may have been confounded with farm-level effects. Larger, farm-replicated studies are required to confirm the present findings.

## Figures and Tables

**Table 1:** Comparison of cortisol concentrations (pg/mg) between husbandry systems and within sampling time.

Husbandry System	Cortisol	Sampling Time			
		T1	T2	T3	T4
Insufficient	Mean	4.7 ^A^	3.75 ^A^	1.01	1.25
	SE	0.47	0.47	0.09	0.19
	CI low	3.78	2.84	0.827	0.88
	CI high	5.61	4.67	1.19	1.63
	*n*	10	10	10	10
Adequate	Mean	3.37	3.51 ^A^	1.07	1.47
	SE	0.25	0.35	0.09	0.16
	CI low	2.87	2.83	0.89	1.16
	CI high	3.87	4.19	1.26	1.78
	*n*	10	10	10	10
Optimal	Mean	2.76 ^B^	1.98 ^B^	1.2	1.49
	SE	0.29	0.19	0.12	0.28
	CI low	2.2	1.61	0.97	0.94
	CI high	3.32	2.36	1.43	2.04
	*n*	10	10	10	10

Data are means ± standard error ^A,B^ mean difference at *p* < 0.01. SE = standard error; CI = 95% confidence interval.

**Table 2:** Quartiles of cortisol concentrations (pg/mg) and the corresponding antibody titre (as reciprocal of serum dilutions).

Quartile	Cortisol					Antibody Titre				
	Mean	SE	CI Low	CI High	CV	Mean	SE	CI Low	CI High	CV
1	0.86	0.07	0.81	0.91	0.16	62.20	1.12	49.70	77.80	0.14
2	1.48	0.05	1.40	1.56	0.15	42.40	1.20	29.70	60.70	0.25
3	2.52	0.17	2.48	2.75	0.14	34.60	1.50	15.50	76.80	0.35
4	4.85	0.52	4.02	4.87	0.26	17.00	1.23	10.70	24.00	0.24

Cortisol concentrations are pg/mg, antibody titres are geometric means of antibody response to RB51 vaccination. SE = standard error; CI = 95% confidence interval; CV = coefficient of variation.

**Table 3:** Linear regression model with cortisol concentrations (log-transformed), sampling time, husbandry system and their interaction as fixed effects, antibody titre (log-transformed) was the dependent variable and the animal was the random intercept.

Fixed Effects	Random Effect	Estimate	SE	*t*-Value	*p*-Value	CI Low	CI High	Marginal *R*^2^	Conditional *R*^2^
Intercept	Animal	3.368	1.545	2.180	0.031	0.340	6.397	0.533	0.689
Cortisol		−3.133	0.905	−3.463	0.001	−4.905	−1.359		
Sampling time		0.904	0.330	2.744	0.007	0.274	1.550		
Husbandry system		0.997	0.570	1.750	0.083	−0.120	2.113		
Sampling time × husbandry system		0.208	0.178	1.168	0.246	−0.141	0.556		

SE = standard error; CI = 95% confidence interval.

## Data Availability

The original contributions presented in this study are included in the article/Supplementary Materials. Further inquiries can be directed to the corresponding author.

## Supplementary Materials

The following supporting information can be downloaded at: https://www.mdpi.com/article/10.3390/vetsci13070612/s1, Figure S1: Hair cortisol concentrations (pg/mg) according to husbandry system and sampling times; Table S1. Logistic regression with cortisol concentrations (log-transformed), sampling time and husbandry system as fixed effects; antibody titre (binary) was the dependent variable.
