# Supplementary material for: The Effect of the Husbandry System and Cortisol Status on the Response of Water Buffalo Calves to Vaccination with the Brucella abortus Vaccine RB51
Source: Vet Sci. 2026 Jun 25;13(7):612. doi: 10.3390/vetsci13070612 (PMC13417067; doi:10.3390/vetsci13070612)
Supplement: Supplementary file 1 [file vetsci-13-00612-s001.zip › Figure S1.pdf]

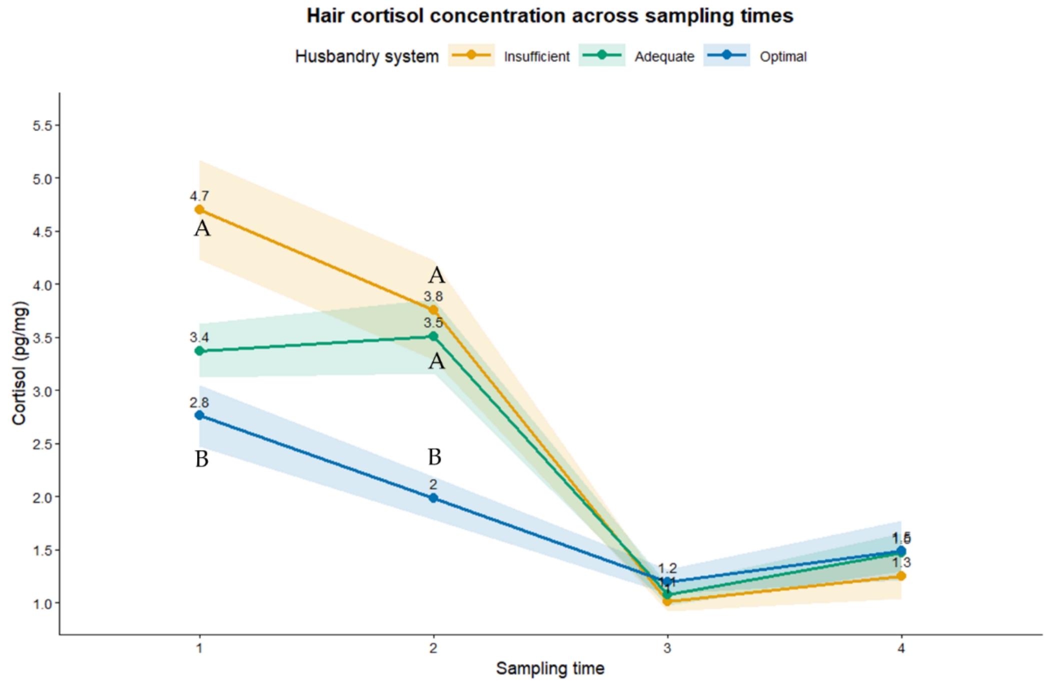

Figure S1. Hair cortisol concentrations (pg/mg) according to husbandry system and sampling times.

[A, B] mean difference at $p < 0.01$
