# Supplementary material for: The Effect of the Husbandry System and Cortisol Status on the Response of Water Buffalo Calves to Vaccination with the Brucella abortus Vaccine RB51
Source: Vet Sci. 2026 Jun 25;13(7):612. doi: 10.3390/vetsci13070612 (PMC13417067; doi:10.3390/vetsci13070612)
Supplement: Supplementary file 1 [file vetsci-13-00612-s001.zip › Table S1.pdf]

Table S1. Logistic regression with cortisol concentrations (log-transformed), sampling time and husbandry system as fixed effects; antibody titre (binary) was the dependent variable

Fixed effects	Odds ratio	CI low	CI high	McFadden pseudo-R^2	AUC (95% CI)	Accuracy
Intercept	0.156	0.017	1.239	0.389	0.872 (0.82–0.92)	0.765
Cortisol	0.194	0.015	2.220			
Sampling time	2.623	1.463	5.154			
Husbandry system	1.361	0.759	2.506			

CI = 95 % confidence interval; AUC (95% CI) = Area under the curve, 95 % confidence interval; Accuracy was computed at the classification threshold of 0.5.
